# Are Female Paraphilias Hiding in Plain Sight? Risqué Male–Male Erotica for Women in Sinophone and Anglophone Regions

**DOI:** 10.1007/s10508-021-02107-4

**Published:** 2021-11-22

**Authors:** Anna Madill, Yao Zhao

**Affiliations:** School of Psychology, University of Leeds, Leeds, LS2 9JT UK

**Keywords:** Boys’ Love, Yaoi, Danmei, Pornography, Paraphilia, DSM-5

## Abstract

Female-oriented male–male erotica is a genre of popular culture often know as Boys’ Love (BL), yaoi, and danmei. It is one of the largest by-and-for women sexual subcultures and a global phenomenon. With the largest data sets in the field, we ask: Which risqué sexual content do Sinophone (Chinese-speaking) and Anglophone (English-speaking) participants particularly enjoy in BL and does this differ between cultures?, and Are there sub-demographics in Sinophone and in Anglophone culture who enjoy particular forms of risqué sexual content in BL and do these forms relate also to enjoyment of particular storylines and concern with legal issues? The material studied meets the DSM-5 definition of the paraphilic, and little is known about paraphilias in women or in the general population. Using Categorical Principal Component Analysis we explored one 15-response question from our Sinophone (*N* = 1922) and Anglophone (*N* = 1715) BL fandom surveys: Which risqué sexual content do you particularly enjoy in BL? We also tested for associations with seven demographic and other BL content-related questions. Notably, the component structure was nearly replicated between the two independent samples, in order of strength: BDSM Specialist, Mechanoid/Animal Sex Specialist, Underage Sex Specialist, and Minority Paraphilia Specialist. In both samples, it was the avid BL fans and/or those who liked explicitly sexual stories, a largely overlapping demographic, who most engage the risqué content, while, for the Sinophone, this included also more non-heterosexual and/or other-gendered people. We conclude that women’s paraphilias have been largely overlooked because they might be expressed more commonly through fantasy than action, that their mass expression has awaited both the means and the market force, and that current conceptualization of, and assumptions about, paraphilias is overly modeled on that of men.

## Introduction

Female-oriented male–male erotica is a genre of popular culture known by several names, predominantly Boys’ Love (BL), yaoi, and, in Mainland China, danmei. BL presents in manga, light novels, anime, video games, fan art, and fan fiction, amongst others. Its defining theme is romantic and sexual relationships between men. Male–male romance appeared in Japanese girls’ manga in the early 1970s. Contemporaneously, Anglophone fan fiction of the TV series Star Trek appeared focusing on a fantasized love affair between the male characters Kirk and Spock (McLelland & Welker, 2015). Female-oriented male–male erotica took off in the Sinophone regions of southeast Asia during the 1980s (Madill, 2020).

BL is now one of the largest by-and-for women sexual subcultures and a truly global phenomenon (Williams, 2015). For example, the danmei website Jinjiang Literature has over 2 million log-ins per day, which is not even required for basic online reading, and a google search on the genre-specific designation “yaoi” gets 121 million results. In this article, we compare engagement with the more risqué content of BL in the Anglophone fandom and the Sinophone fandom of the greater China area (Mainland China, Hong Kong, Macau, and Taiwan). These are regions of world importance but of very different cultural outlook and sociopolitical context. We argue that interest in male–male sexuality is a common female paraphilia through which women engage a range of more recognized paraphilic themes. We posit that women’s atypical sexual interests may have been largely overlooked because paraphilias are conceptualized in terms of how they present in men.

The first research reporting demographic information on the BL fandom in our regions of interest is Chang (2007: Taiwan), Zhao (2008: Mainland China), and Pagliassotti (2008: English-speaking and Italian-speaking European Union). Our own research confirms that the demographic overwhelmingly consists of women although, in contrast to Sinophone regions, the engaged Anglophone demographic includes more men (male: Sinophone 11%, Anglophone 16%), people with a wider range of sexual orientations and lower rates of heterosexual identification (non-heterosexual: Sinophone 34%, Anglophone 69%), and a wider and older age range (Sinophone range 12–37 years, mean 20 years; Anglophone range 11–70 years, mean 23 years) (Madill & Zhao, 2021b). We have also provided novel evidence that a broad demographic of young people in Sinophone regions is familiar with BL as a casual interest in contrast to Anglophone regions where it appears more an intense and niche pass-time (Madill & Zhao, 2021c).

Paraphilias are defined in the Diagnostic and Statistical Manual of Mental Disorders (DSM-5: APA, 2013) as intense and frequently engaged atypical sexual interests. Sexually arousing contexts might include children, non-consenting adults, inanimate objects, and the suffering or humiliation of self or others (Brown, 2019). Salmon and Symons (2003) suggest that women with an interest in male–male sexuality may have a “psychosexual quirk” analogous to male paraphiliacs. Interestingly, the benchmarking to men here implies an othering of women’s sexuality and a potential blind spot in the field. The only other reference to paraphilias and BL we can find is by Lindqvist and Carlström (2020) who explore the possibility that autoandrophilia, the propensity of a female to be sexually aroused by the thought of herself as male, might explain at least some of the interest in BL. However, they criticize the invoking of a paraphilia in the context of BL because it undermines trans BL fan identity.

We believe it is still fruitful to consider interest in male–male sexuality a common, yet overlooked, female paraphilia and that autoandrophilia does not exhaust BL’s paraphilic potential. However, there is understandable sensitivity to potentially pathologizing labels (Joyal, 2021), and the BL community is already stigmatized within fandom and under increasing legal scrutiny. It is therefore important to note that in the DSM-5, paraphilias are considered part of the normal diversity of human sexuality and are helpfully distinguished from paraphilic disorders that cause distress, problems functioning, and/or harm (APA, 2013).

Little is known about paraphilias in women, or in the general population, and most knowledge has been gleaned from sexual offenders and clinical populations (Yakeley & Wood, 2014). In a rare study of paraphilic interest in the general population, Canadian men reported significantly less repulsion and more arousal to paraphilic acts than did women, with sex drive accounting for the sex difference (Dawson et al., 2016). The limited and dated research on women’s paraphilic interest suggests that the most common female paraphilia may be sexual masochism (e.g., Levitt et al., 1994). However, perhaps researchers have been looking in the wrong place and would be better browsing the TV listings. Today, sexually inflected male friendships are not uncommon in mainstream entertainment and are used knowingly, such as in the series Sherlock, to attract a large and ardent female audience (Abad-Santos, 2014). In China, the effect is staggering and, for example, despite poor production values, by the summer of 2018 the live action adaption of danmei novel Guardian had accumulated 25 billion online plays*.*

There is little or no comparative research on the content of BL in Anglophone and Sinophone regions. One exception is Madill et al.’s (2018) comparison of Harry Potter fan fiction, the authors concluding that the stories tend to mirror the relative social conservatism and liberalism of their cultures of origin. There is a blanket ban on sexually explicit material in Mainland China, except in high literature or classical arts, and Williams (2020) suggests this has influenced danmei writers to produce romantic or only gently suggestive stories to avoid censorship (see also Zhao & Madill, 2018; Madill & Zhao, 2021a). Moreover, all formal publications must have a government permit, meaning that most self-published material, as is most danmei, is illegal in this regard. Hence, engagement with danmei in Mainland China has potentially serious consequences and since 2011, there have been several high profile arrests within the community for spreading pornographic materials online (see Yang & Xy, 2016). In Anglophone countries, engaging with BL is not without risk and material may, for example, be considered to contravene child pornography laws in some jurisdictions (Madill, 2015).

Our focus in this article is engagement with, what we label in our survey, the risqué sexual content of BL, comparing Sinophone and Anglophone fandoms. We used the term “risqué” because it is likely familiar to the demographic of interest and to have non-pathologizing connotations. We base our identification of risqué content on our familiarity with BL, the debates within BL fandom, the global legislative and moral environment with regard to pornographic materials, and feedback from our piloting. Hence, we provided the following 13 options for our survey question, Which risqué sexual content do you particularly enjoy in BL?: animal–animal sex (including humanoid beings with animal features); BDSM (bondage and discipline, dominance and submission, sadism and masochism); binding; group sex; human–animal sex (including humanoid beings with animal features); human–mechanoid sex (e.g., robot and android); mechanoid–mechanoid sex; rape; role play sex; sex toys; tentacle sex; underage sex (both partners underage); and underage sex (one underage and one older partner). We also included “none” and an optional free-text response to “other” to capture content we had not listed.

To clarify, first, in BL binding can be used as a visual aesthetic which enhances and sexualizes parts of the body and is not always portrayed alongside other BDSM practices such as those involving restriction of movement. We therefore included binding as an independent option. Second, due to the complexity of the format, our survey included a separate multiple response question on incest material: Which “incestual” sexual relationships do you particularly enjoy in BL? (Select all that apply or leave blank if you don’t particularly enjoy incestual sexual relationships in BL). Participants were able to select as many of the response options as they wanted from: father/son (seme/uke, i.e., “top/bottom”), son/father (seme/uke), brothers, uncle/nephew (seme/uke), nephew/uncle (seme/uke), each of which had four sub-types: blood, adoptive, step, and in-law (i.e., in total 20 options) (see Madill & Zhao, 2021b). Although incest material is risqué in the same sense as the material analyzed in this article, the clinical literature tends to consider incest per se separately to paraphilias where it is most likely subsumed under sexual interest in children (Craig & Bartels, 2021). We will compare our analysis of the risqué content and incest material data in the Discussion.

We address two research questions: Which risqué sexual content do Sinophone and Anglophone participants particularly enjoy in BL and does this differ between cultures?, and Are there sub-demographics in Sinophone and in Anglophone culture who enjoy particular forms of risqué sexual content in BL and do these forms relate also to enjoyment of particular storylines and concern with legal issues? Addressing these questions has potential for novel insights into the BL fandom and, more generally, into paraphilias in women and in the general population of two culturally diverse regions of global importance.

## Method

The survey and methodology were approved by the Research Ethics Committee of the School of Psychology, University of Leeds, UK. Ethical approval included the principle that submission of a completed survey to a researcher, or online, constituted informed consent. No individual participant is identifiable in this article.

### Participants

The Sinophone survey *N* = 1922 and the Anglophone survey *N* = 1715, the latter including only participants who noted their first language to be English. Nationalities given in the Sinophone survey are: Chinese (*N* = 1883), Hong Kongese (*N* = 38), and Taiwanese (*N* = 1). Countries of current residence are: Mainland China (*N* = 1697, including Macau *N* = 4), Hong Kong (*N* = 198), UK (*N* = 12), USA (*N* = 5), and other (*N* = 10). Nationalities given in the Anglophone survey are: American (*N* = 757), British (*N* = 335), Australian (*N* = 74), Canadian (*N* = 66), dual nationality of two of the aforementioned countries (*N* = 6), New Zealander (*N* = 12), Asian nationalities (*N *= 135), other European nationalities (*N* = 30), Central/South American nationalities (*N* = 14), African nationalities (*N* = 6), and unknown (*N* = 280: e.g., “white,” “Hispanic”). Countries of current residence are: America (*N* = 1109), UK (*N* = 334), Australia (*N* = 96), Canada (*N* = 83), Singapore (*N* = 25), Philippines (*N* = 20), New Zealand (*N *= 14), Japan (*N* = 9), Malaysia (*N *= 7), Italy (*N* = 3), South Africa (*N* = 3), China (*N* = 2), India (*N* = 2), United Arab Emirates (*N* = 2), Bangladesh, Germany, Spain, Sweden, Zambia (1 each; *N* = 5), and unknown (*N* = 1). Our survey asked “What is your gender?” and provided the responses “male,” “female,” and “other.” Free-text responses to “other” include such descriptions as “gender fluid,” “agender,” and “neutral” (for full list see, Madill & Zhao, 2021c). By gender, the Sinophone sample is 11% male, 88% female, and 1% other-gendered; the Anglophone sample 16% male, 75% female, and 9% other-gendered.

### Measures

Our 43-question survey has five sections: demographics, BL materials, feelings about BL, social relationships, and other erotic materials (including engagement with heterosexual, lesbian, and pornography for gay men). Responses are, in the main, on a five-point Likert scale, and some questions include a free-text response box. The English-language version opened in November 2014 and was promoted via relevant internet forums, social network websites, and by e-mailing anime and manga clubs. The version in Simplified Chinese opened in March 2015 and was promoted via universities in China, leaflets distributed with products from an online Chinese BL shop, and manga events in China at which a paper copy was provided for immediate completion and transferred online for analysis (*N* = 200). Data were collected until November 14, 2018.

Responses to What is your sexual orientation? were recoded to two categories—heterosexual and non-heterosexual. The latter includes responses bisexual, homosexual/lesbian/gay, not sure, and other (Sinophone survey) and bisexual, homosexual/lesbian/gay, polysexual/pansexual, not sure, and other (Anglophone survey). This managed the fact that polysexual/pansexual was not included in the Sinophone survey because there is active discouragement of non-heterosexuality in Mainland China and no common-use Chinese translation of these terms. We also asked If you selected Other, please specify. Specified other sexual orientations include asexual, demisexual, and queer (for full list, see Madill & Zhao, 2021c). Of the Sinophone participants, 56% of the men, 55% of the women, and 25% of the other-gendered people are heterosexual. Of the Anglophone participants, 10% of the men, 39% of the women, and 3% of other-gendered people are heterosexual.

### Procedure

To address research question Which risqué sexual content do Sinophone and Anglophone participants particularly enjoy in BL and does this differ between cultures?, we analyzed the multiple response survey question, Which risqué sexual content do you particularly enjoy in BL? Participants could select as many of the response options as they wanted (see Introduction). Hence, each option was coded as an individual binary-nominal type question (1 = no, 2 = yes). Two additional options were provided, the latter with an optional free-form text box: “none of the above” and “other.”

Categorical principal component analysis (CATPCA) was used to inspect the underlying structure in the form of components for each cultural group. CATPCA is a data reduction technique used when the data are categorical, or for variables of mixed measurement, and does not assume a linear relationship between data (Manisera et al., 2010). To perform CATPCA, the Categories Module was applied. This reduces a data set consisting of many variables with complicated correlational pattern to a smaller number of relatively uncorrelated components each of which consists of relatively highly inter-correlated variables. Deciding the most meaningful number of components involves consideration of eigenvalue which indicates the amount by which a component has been stretched in a geometrical transformation. Eigenvalue by component and percentage variance accounted for (PVAF) are the most important indictors of fit for the principle components (i.e., most meaningful reduction of the data set with regard to semi-independent inter-correlations of variables) (Linting & van der Kooij, 2012).

To address research question Are there sub-demographics in Sinophone and in Anglophone culture who enjoy particular forms of risqué sexual content in BL and do these forms relate also to enjoyment of particular storylines and concern with legal issues?, we explored if a sub-demographic is associated with each risqué sexual content CATPCA component within each cultural group. Demographic variables of interest are gender, sexual orientation, and fandom intensity. Participants were identified who had endorsed all of the highest loading, i.e., key variables, on each of the components defined as 0.5 and above (Table 4). For example, to be classified as a BDSM Specialist, an Anglophone participant needed to have endorsed all four key variables BDSM, sex toys, binding, and role play sex. First, using endorsement of key variables, we calculated the percentage of Sinophone and Anglophone participants engaging with each CATPCA risqué sex component by gender and sexual orientation. Second, the Chi-square test of independence was used to determine whether any of two categorical demographic variables—gender (male, female, other), sexual orientation (heterosexual, non-heterosexual)—are associated with each risqué sex component within cultural group. We also explored the extent to which any sub-demographic associated with each risqué sexual content CATPCA component within each cultural group cohered in terms of fandom intensity, liking for BL sexual storyline type and concern over legal issues with regard to sexual content. Hence, third, because the data are not normally distributed, the nonparametric Mann–Whitney U test was used to test for differences in median on the Likert scale variables—How intensely are you a BL fan?, I like BL stories that are mostly romance and no sex, I like BL stories with “implicit relationships,” I like BL stories with explicit sex, and To what extent do you worry about legal issues in relation to the sexual content of BL?—within cultural group in relation to endorsement of each risqué sex component. The result of all comparisons undertaken is reported, and, where significant, effect size is provided—Cramer’s V (Chi-square) and Pearson’s r (Mann–Whitney)—where a trivial effect is less than 0.1, a small effect 0.1–0.29, a medium effect 0.3–0.49, and large effect 0.5 and above (Cohen, 1988). The demanding significance level of *p* < 0.001 was set throughout.

## Results

### Frequency Endorsement of Risqué Sexual Content Variables

**Fig. 1:**
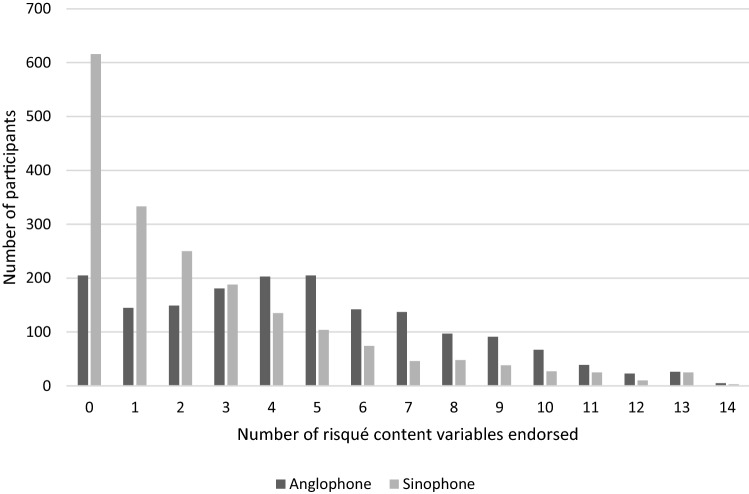
Frequency endorsement of risqué content variables by group

The survey provided 13 risqué content options plus none and other. Where other and the 13 content options count as one endorsement each, the Sinophone participants endorsed a mean of 2.59 options with none/0 = 616, and the Anglophone participants endorsed a mean of 4.76 options with none/0 = 205 (Figure 1). The pattern in the Sinophone sample is an exponential decrease in the number of options endorsed, while the Anglophone pattern is bimodal at 0 and 5. Anglophone participants typically endorse more options that do Sinophone and do so on the risqué sexual content question (see also Bhugra et al., 2010). However, this does not affect the with-group demographic patterns identified.

### CATPCA of Risqué Sexual Content—Sinophone Data

Kaiser’s criterion is the most common technique in deciding the number of meaningful components, and it recommends retaining all with an eigenvalue above 1 (for alternatives, see Velicer et al., 2000). Auerswald and Moshagen (2019) indicate that the Kaiser criterion is reliable when sample size is above *N* = 500, and the Sinophone sample is *N* = 1922. This means that the decision was made to retain components 1–4. (Component 5 has an eigenvalue of 0.897.)

The total PVAF across the four components accounts for 58.65% of total variance in response of the Sinophone participants to Which risqué sexual content do you particularly enjoy in BL? (Table 1). This is very close to the 60% of explained variance suggested for a “good” factor analysis by Hair et al. (2018). Unrotated component 1 accounts for the largest PVAF at 34.57%, with components 2–4 accounting, respectively, for 9.23%, 8.03%, and 6.81%. Cronbach’s alpha is a measure of internal consistency, with a coefficient of 0.70 or higher considered acceptable in the social sciences (UCLA: Statistical Consulting Group, 2020). So unrotated, on this criterion, only component 1 has good internal consistency.

**Table 1:** Variance accounted for in the four components retained in the Sinophone responses to which risqué sexual content do you particularly enjoy in BL?

Component	Unrotated solution			Rotated solution^a^		
	Cronbach’s alpha	Eigenvalue	PVAF	Cronbach’s alpha	Eigenvalue	PVAF
1	.86	5.19	34.57	.84	3.77	25.12
2	.30	1.38	9.23	.74	2.12	14.15
3	.18	1.21	8.03	.72	1.84	12.25
4	.02	1.02	6.81	.09	1.07	7.13
Total	.95^b^	8.80	58.65	.95	8.80	58.65

^a^Rotation method: Varimax with Kaiser normalization

^b^Total Cronbach’s alpha is based on the total eigenvalue

Without changing fit, an orthogonal, varimax rotation can produce the most structurally simple solution in which each variable loads as highly as possible on only one component. A simplified solution was, indeed, produced through rotation for the Sinophone data. In the rotated solution: (i) all four components retain an eigenvalue above 1; (ii) PVAF is redistributed in such a way as to maintain the strength of component 1 while improving that of the three smaller components: 25.12%, 14.15%, 12.25%, and 7.13%, respectively; and (iii) Cronbach’s alpha is now above 0.7 for components 1, 2, and 3 indicating that all three now have an acceptable level of internal consistency. In the rotated solution, component 4 still has low internal consistency, but, despite this, the variable loadings, as described below, allow component 4 to be interpreted meaningfully.

Loading of each variable on each component is indicated by the Pearson correlation, the value of which ranges between -1 and 1. Within context, variable none can be interpreted as “no particular enjoyment of risqué content in BL.” Components derived from CATPCA are interpretable in light of the variables loading most highly on each (Table 2). The variables loading most highly on component 1 are: BDSM, binding, group sex, rape, role play sex, sex toys, tentacle sex, and a negative loading for none. The thematic similarity of the variables loading on component 1 suggests that it denotes a BDSM Specialist enjoyment in risqué sexual content in BL. The variables leading most highly on component 2 are: animal–animal sex, human–animal sex, human–mechanoid sex, and mechanoid–mechanoid sex. The thematic similarity of these variables, and high loading of human–animal sex on component 2, leads to the decision to include human–animal sex in component 2 even though it has a very slightly higher loading in component 1. The variable loadings suggest that component 2 strongly denotes a Mechanoid/Animal Sex Specialist enjoyment in risqué sexual content in BL. The variables loading most highly on component 3 are: underage sex (both) and underage sex (one). This suggests that component 3 strongly denotes an Underage Sex Specialist enjoyment in risqué sexual content in BL. Even though Cronbach’s alpha (0.087) indicates low internal consistency, the fact that the only variable loading highly—and it loads extremely highly—on component 4 is other, makes this component interpretable meaningfully. That is, component 4 is interpretable as denoting a Minority Paraphilia Specialist enjoyment in risqué sexual content in BL, but in ways not covered by the options provided in the survey.

**Table 2:** Comparison of rotated component structure of Sinophone and Anglophone risqué sexual content CATPCAs

Variable	Sino	Anglo	Sino	Anglo	Sino	Anglo	Sino	Anglo
	1 BDSM Specialist		2 Mechanoid/Animal Sex Specialist	2 Animal Sex Specialist	3 Underage Sex Specialist		4 Minority Paraphilia Specialist	
Animal–animal sex	.43	.15	.51	.81	.16	.14	.00	.10
BDSM	.60	.77	.17	− .02	.15	.07	− .08	.08
Binding	.75	.68	.05	.05	.16	.07	− .05	.11
Group sex	.53	.47	.04	− .08	.43	.20	.00	.35
Human–animal sex	.48	.12	.47	.81	.13	.22	.06	.12
Human–mechanoid sex	.14	.18	.83	.41	.09	− .02	.01	.64
Mechanoid–mechanoid sex	.07	.12	.81	.36	.15	− .03	.01	.72
Rape	.65	.30	− .07	− .06	.36	.50	.00	.25
Role play sex	.61	.62	.25	.23	− .10	.10	.05	− .02
Sex toys	.72	.73	.19	.15	.14	.05	− .08	.09
Tentacle sex	.60	.37	.19	.19	.28	.07	− .03	.44
Underage sex (both)	.11	.11	.22	.21	.82	.82	− .03	.03
Underage sex (one)	.23	.12	.14	.19	.79	.86	.03	− .01
None	− .64	− .64	− .26	− .14	.00	− .23	− .37	− .02
Other	− .06	− .10	.00	− .20	.01	.14	.95	.57

Participants indicating other were invited to provide a free-text explanation of their response (*N* = 49; 2.5%): depends on the story and how author present the story (*N* = 9), ABO [Alpha/Beta/Omega: a fanwork kink trope in which, amongst other characteristic themes, Alphas are dominant and can impregnate Omegas, while Betas are subordinate to Alphas] (*N* = 5), aphrodisiacs (*N* = 4), cross-dressing (*N* = 4), gekokuj [meaning that the person who is normally in the more submissive position takes over the dominant role] (*N* = 4), younger dominant and older submissive (*N* = 4), special scenario (*N* = 3), uniform play (*N* = 3), group sex with one submissive (*N* = 2), human–ghost relationship (*N* = 2), sex during male pregnancy (*N* = 2), special relationship, such as teacher–student, or supervisor and subordinate (*N* = 2), unfaithful relationship (*N* = 2), and one each for omorashi [arousal from having a full urinary bladder or a sexual attraction to someone experiencing this feeling], one character plays both dominant and submissive, and incest (*N* = 3).

### CATPCA of Risqué Sexual Content—Anglophone Data

The Kaiser criterion is reliable when simple size above *N* = 500 and the Anglophone sample is *N* = 1713. Hence, as with the Sinophone data, the decision was made to retain components 1–4. (Component 5 has an eigenvalue of 0.963.) The total PVAF across the four components account for 55.63% of total variance in response of the Anglophone participants to Which risqué sexual content do you particularly enjoy in BL? (Table 3). This is a little lower than the Sinophone CATPCA, but reasonably close to the 60% of explained variance suggested for a good factor analysis. Unrotated component 1 accounts for the largest PVAF at 28.56%, with components 2–4 accounting, respectively, for 10.49%, 9.59%, and 6.98%. Cronbach’s alpha indicates that although four components are retained on the basis of eigenvalue, unrotated only component 1 has good internal consistency.

**Table 3:** Variance accounted for in the four components retained in the Anglophone responses to Which risqué sexual content do you particularly enjoy in BL?

Component	Unrotated solution			Rotated solution^a^		
	Cronbach’s alpha	Eigenvalue	PVAF	Cronbach’s alpha	Eigenvalue	PVAF
1	.82	4.28	28.56	.77	2.94	19.57
2	.39	1.57	10.49	.66	1.87	12.46
3	.33	1.44	9.59	.64	1.86	12.37
4	.05	1.05	6.98	.62	1.68	11.22
Total	.94^b^	8.34	55.63	.94	8.34	55.63

^a^Rotation method: Varimax with Kaiser normalization

^b^Total Cronbach’s alpha is based on the total eigenvalue

A simplified solution was produced through rotation for the Anglophone data. In the rotated solution: (a) all four components retain an eigenvalue above 1; (b) PVAF is redistributed in such a way as to maintain the strength of component 1 while improving that of the three smaller components: 19.57%, 12.46%, 12.37%, and 11.22%, respectively, and (c) Cronbach’s alpha remains above 0.7 for components 1 and improves to just below 0.7 for components 2, 3, and 4 indicating reasonable levels of internal consistency.

Components derived from CATPCA are interpretable in light of the variables loading most highly on each (Table 2). The variables loading most highly on component 1 are: BDSM, binding, group sex, role play sex, sex toys, and a negative loading for none. The thematic similarity of the variables loading on component 1 suggests that it denotes a BDSM Specialist enjoyment in risqué sexual content in BL. The variables leading most highly on component 2 are: animal–animal sex and human–animal sex. This suggests that component 2 strongly denotes an Animal Sex Specialist enjoyment in risqué sexual content in BL. The variables loading most highly on component 3 are: rape, underage sex (both), and underage sex (one). This suggests that component 3 denotes an Underage Sex Specialist enjoyment in risqué sexual content in BL. The variables loading most highly on component 4 are: human–mechanoid sex, mechanoid–mechanoid sex, tentacle sex, and other. This suggests that component 4 denotes a Minority Paraphilia Specialist enjoyment in risqué sexual content in BL.

Participants indicating other were invited to provide a free-text explanation of their response (*N* = 88; 5%): mild forcefulness, SM-lite, forced waiting, pet play sex, bondage without violence or strongly against one’s will (*N* = 14), other animal and non-human related sex including mythological creatures, aliens, vampires, werewolves or fairies (*N* = 9), depends on the stories or authors (*N* = 8), blood, violence, and gore/guro [eroticism and the grotesque] (*N* = 7), the meaning of “underage” (*N* = 5), special kind of relationship such as teacher–pupil (*N* = 5), liking almost everything (*N* = 4), special scenario such as public sex (*N* = 4), food play sex (*N* = 3), dominant becoming a subordinate (*N* = 3), ABO (see above, *N* = 2), aphrodisiacs (*N* = 2), feet (*N* = 2), fetish (*N* = 2), first time (*N* = 2), fisting (*N* = 2), male pregnancy (*N* = 2), and one each for arse pissing, dirty talk, fellatio, gender bending, humiliation, laboratory-based sex, masturbation, omorashi (see above), sex in water, switching, triangular relationship, and voyeurism (*N* = 12).

### Sub-demographics Associated with CATPCA Components

To explore whether a sub-demographic of each data set is associated with each risqué sexual content CATPCA component, participants were identified who had endorsed all of the highest loading, key variables on each of the components (Table 4). As explained above, participants endorsing all key variables defined as a loading of 0.5 and above of a component were identified and the percentage of the Sinophone and Anglophone sample engaging with each CATPCA risqué sex component reported by gender and sexual orientation (Table 5). Using this method, a higher percentage of Anglophone participants are risqué sexual content specialists than Sinophone participants: 23.7% compared to 4.0% for BDSM Specialist, 22.2% compared to 5% for Animal Sex Specialist, and 12.1% compared to 6% for Underage Sex Specialist. The exception is Minority Paraphilia Specialist which is engaged with by 7.6% of the Sinophone sample but only 1.5% of the Anglophone. Notably, while percent engagement of the Anglophone sample decreases with decline in component strength, in the Sinophone sample it gently increases.

**Table 4:** Key variables in order of importance for each risqué sexual content CATPCA component by culture used to identify engaged demographic

Component	BDSM Specialist	Mechanoid/Animal Sex Specialist	Underage Sex Specialist	Minority Paraphilia Specialist
Sinophone key variables	Binding Sex toys Rape Role play sex Tentacle sex BDSM Group sex	Human–mechanoid sex Mechanoid–mechanoid sex Animal–animal sex	Underage sex (both) Underage sex (one)	Other
Anglophone key variables	BDSM Sex toys Binding Role play sex	Human–animal sex Animal–animal sex	Underage sex (one) Underage sex (both) Rape	Mechanoid–mechanoid sex Human–mechanoid sex Other

**Table 5:** Percentage of Sinophone and Anglophone BL fandom engaging with each CATPCA risqué sex component by gender and sexual orientation

C^a^	% of Sinophone (*N* = 1922)						% of Anglophone (*N* = 1715)					
	Total	Male	Female	Other	Het	Non-het	Total	Male	Female	Other	Het	Non-het
1	4.0% (*N* = 77)	0.4% (*N* = 8)	3.4% (*N* = 65)	0.2% (*N* = 4)	1.5% (*N* = 29)	2.5% (*N* = 48)	23.7% (*N* = 406)	3.4% (*N* = 59)	17.7% (*N* = 303)	2.6% (*N* = 44)	5.9% (*N* = 101)	17.8% (*N* = 305)
2	5.0% (*N* = 97)	0.3% (*N* = 6)	4.5% (*N* = 86)	0.3% (*N* = 5)	1.8% (*N* = 35)	3.2% (*N* = 62)	22.2% (*N* = 380)	4.0% (*N* = 68)	16.2% (*N* = 277)	2.0% (*N* = 35)	6.1% (*N* = 104)	16.1% (*N* = 276)
3	6.0% (*N* = 115)	1.2% (*N* = 23)	4.5% (*N* = 87)	0.3% (*N* = 5)	2.1% (*N* = 41)	3.9% (*N* = 74)	12.1% (*N* = 207)	1.9% (*N* = 32)	9.1% (*N* = 156)	1.1% (*N* = 19)	3.2% (*N* = 55)	8.9% (*N* = 152)
4	7.6% (*N* = 146)	0.9% (*N* = 18)	6.6% (*N* = 127)	0.05% (*N* = 1)	5.3% (*N* = 101)	2.3% (*N* = 45)	1.5% (*N* = 25)	0.3% (*N* = 5)	0.93% (*N* = 16)	0.2% (*N* = 4)	0.3% (*N *= 5)	1.2% (*N* = 20)

^a^Component

The Chi-square test of independence was used to determine whether any of two categorical demographic variables—gender (male, female, other), sexual orientation (heterosexual, non-heterosexual)—are associated with each of the four components within cultural group. The Mann–Whitney was used to test for differences in median on the Likert scale variables—How intensely are you a BL fan?, I like BL stories that are mostly romance and no sex, I like BL stories with “implicit relationships,” I like BL stories with explicit sex, and To what extent do you worry about legal issues in relation to the sexual content of BL?—within cultural group in relation to endorsement of each of the four components (Table 6).

**Table 6:** Summary statistical results for Sinophone and Anglophone demographic, storyline, and legal concern associated with each CATPCA risqué sex component

Component	Variables	Sinophone		Anglophone	
		Effect size	Direction	Effect size	Direction
BDSM Specialist	Gender	Small^a^	Male↓ female↓ other↑	/	/
	Sexual orientation	Small	Het↓ non-het↑	Trivial	Het↓ non-het↑
	Fandom intensity	Small	↑	Large	↑
	Romance no sex	/^b^	/	/	/
	Implicit relationship	/	/	/	/
	Explicit sex	Small	↑	Small	↑
	Legal concern	/	/	/	/
Mechanoid/Animal Sex Specialist	Gender	Small	Male↓ other↑	/	/
	Sexual orientation	Small	Het↓ non-het↑	/	/
	Fandom intensity	Small	↑	Small	↑
	Romance no sex	/	/	/	/
	Implicit relationship	/	/	/	/
	Explicit sex	Small	↑	Small	↑
	Legal concern	Small	↑	/	/
Underage Sex Specialist	Gender	Small	Male↑ female↓ other↑	/	/
	Sexual orientation	Small	Het↓ non-het↑	/	/
	Fandom intensity	Small	↑	Small	↑
	Romance no sex	Trivial	↓	/	/
	Implicit relationship	/	/	/	/
	Explicit sex	Small	↑	Small	↑
	Legal concern	/	/	/	/
Minority Paraphilia Specialist	Gender	/	/	/	/
	Sexual orientation	/	/	/	/
	Fandom intensity	/	/	/	/
	Romance no sex	/	/	/	/
	Implicit relationship	/	/	/	/
	Explicit sex	/	/	/	/
	Legal concern	/	/	/	/

^a^Cramer’s V of .090 considered a small effect due to restricted sample size of *N* = 77

^b^/ means not significant

The Minority Paraphilia Specialist is not associated with a clear demographic in our study and may consist of a non-cohesive group of individuals with a variety of niche sexual interests. On the other hand, there is a consistent pattern for the larger three components across the two cultures. Sinophone participants engaging with risqué BL content are likely to be other-gendered, non-heterosexual, avid fans, and/or to like explicit sex stories. In addition, women are less likely to be a BDSM Specialist or Underage Sex Specialist, while men are more likely to be an Underage Sex Specialist and less likely to be a Mechanoid/Animal Sex Specialist. The pattern is very straightforward for Anglophone participants engaging with risqué BL content who are, as the Sinophone, likely to be avid fans and/or to like explicit sex stories.

Variables “fandom intensity” and “like explicit sex stories” were found to be strongly correlated within both cultural groups at *p* < 0.001 (medium effect size). The question, To what extent do you worry about legal issues in relation to the sexual content of BL?, where 1 is low and 5 is high, had the following mean, median, and mode: Sinophone 2.84, 3, 2; Anglophone 1.82, 2, 1.

## Discussion

The most notable result is the near replication of component structure between Sinophone and Anglophone BL fan engagement with risqué sexual content. Hence, we provide robust and novel evidence of a characteristic pattern of dominant paraphilic interests within the overarching vehicle of female-oriented male–male erotica in two major regions of the world, while identifying also some important cultural differences (Zgourides, 2020). Our analysis reveals three well-known paraphilic themes: BDSM, animals/inanimate humanoids, and children. Although four distinct components are extractable, there are few negative variable loadings on each and, where these exist, they are small. This indicates in line with research on paraphilias that atypical sexual interests tend to correlate (Dawson et al., 2016).

### Similarities between the Sinophone and Anglophone groups

For both cultural groups, the BDSM Specialist component accounts for the largest proportion of variance. This is commensurate with BDSM being quite common in BL (Madill, 2016) although, in contrast to its more usual “hard core” connotations, BDSM is often used with humor and as a narrative device to enhance romance and intimacy (Arunrangsiwed et al., 2018; Santos, 2020). The global popularity since 2011 of the book and film Fifty Shades of Grey, written by a women and originally a Twilight fan fiction, may have helped extend the concept of BDSM for a mainstream female audience (Cruz et al., 2019). Hence, the fact that BDSM Specialist is the largest component for both cultures reflects the apparently increasing acceptability of “BDSM-lite” erotica for young women. However, fictional BDSM may also be the least legally problematic of the components.

Animal Sex Specialist accounts for the second largest proportion of variance for both cultural groups, along with Mechanoid Sex for the Sinophone. Anthropomorphism is a common visual style in Japanese manga and anime and the sexualized material it inspires (Egilsdóttir, 2016). Hence, animal elements, such as humanoids with animal ears or tails, are not uncommon in BL and stories can include mythological or supernatural themes with non-human agents (Zhao & Madill, 2018). Animal elements can increase the cuteness and lovability of characters, connote “animal passion,” and is usually very different from portrayal of real sexual acts between humans and animals which is considered extreme pornography and illegal in many jurisdictions (Vetter et al., 2020). In relation to the Sinophone component structure, “mecha” is a popular genre in manga and anime and its erotic potential is leveraged in BL, often in stories that explore the affectional possibilities of humanoid agents. In contrast, the real sex doll and sex robot market caters almost exclusively to men, with concern that it promotes sexual objectification of others (Döring, 2021).

Underage Sex Specialist accounts for the third largest proportion of variance in the data for both cultural groups. In regions where there is no blanket ban on pornography, some distinction is usually made in law between real and virtual child pornography and it is not clear if any BL contravenes current rulings (Madill, 2015). BL fandom is split on the acceptability of sexual elements in material containing young protagonists (Zanghellini, 2009), and this is complicated by the fact that assumptions regarding, and the implications of “underage sex” are different across the two regions studied. In Anglophone countries, age of consent ranges from 16–18 years (Dauda, 2009). On the other hand, age of consent tends toward the lower side in Sinophone regions, being 16 years in Taiwan and Hong Kong (ROC Criminal Law, Article 227, 227–1 229–1; https://www.hklii.org/eng/hk/legis/ord/200/s124.html) and 14 years in Mainland China (https://2009-2017.state.gov/j/drl/rls/hrrpt/2009/eap/135989.htm).

Finally, Minority Paraphilia Specialist accounts for the least proportion of variance for both cultural groups. The Sinophone version loaded almost entirely on other, whereas the Anglophone version loaded heavily also on mechanoid and tentacle sex (see below). We surmise that Minority Paraphilia Specialist consists of a variety of uncommon tastes with little consistency other than being niche interests (Rosen, 2020).

Common to the largest three components is the observation that the paraphilic themes they represent likely present in unexpected ways. That is, BDSM elements are often portrayed with humor, zoophilic/inanimate elements as cute and clearly fantastical humanoids, and hebephilic/pedophilic elements ambiguously by being both fictional and visually stylized (Madill, 2015). This raises the question as to whether paraphilias in women have been largely overlooked because they often take a different form to that of men. Specifically, as frequent, intense, sexually arousing *fantasies* or *behaviors* of an unusual nature, it could be that women’s paraphilias have avoided scrutiny though manifesting more often in imagination than in action. This is in line with the bulk of research that suggests biological and social factors intersect such that men are more likely than women to be interested in physical sexuality (Bhugra et al., 2010) and to engage in risky and sensation-seeking sexual behavior (Marshall, 2007). It may, therefore, be no surprise that BL has flourished alongside the internet which allows women to share unusual sexual interests anonymously en masse. Moreover, as women’s social and economic power has increased, they have gained market force and become innovators of popular entertainment.

### Differences between the Sinophone and Anglophone groups

The differences between the Sinophone and Anglophone samples are the placing within component structure of the variables rape, human–mechanoid sex, mechanoid–mechanoid sex, and tentacle sex.

#### The Variable Rape

The World Health Organization (2012) defines rape as a type of sexual assault in which an offense is committed through performing sexual intercourse or other forms of sexual penetration without the consent of the other person. The act may be carried out by physical force, other form of coercion, and/or as an abuse, and the victim may be unconscious, incapacitated, and/or be unable to offer legitimate consent due to intellectual disability or by being below the legal age of consent (i.e., statutory rape). Rape is a relatively common, but controversial, theme in BL, presented through physical and/or psychological coercion and unclear consent (Frennea, 2011). However, some BL researchers argue that rape is explored in BL as sexual fantasy, and as a plot device to progress the narrative, and has little or no real-world implication (McLelland & Yoo, 2007; Nagaike, 2003).

That rape appears in Sinophone BDSM Specialist but in Anglophone Underage Sex Specialist is interpretable in terms of cultural differences. Whereas in both Anglophone and Sinophone regions statutory rape is legislated, i.e., automatic under the age of consent, it appears less recognized in Mainland China by offenders and the victim’s family and is relevant only to girls. That is, reports are that there were 3174 child victims of sexual assault (associated with only 1779 cases) between 2013 and 2017 (Girls’ Protection Fund, 2018; Xu, 2019). Families are likely to make settlement in private, which accounts for the relatively low number of cases recorded. Thus, although sexual assault below the age of consent is given increasing attention in China (Lyu et al., 2020), the connection between underage sex and rape, particularly of boys, is not strong in the public mind.

#### The Variables Human–Mechanoid Sex and Mechanoid–Mechanoid Sex

Science fiction originated in Anglophone countries (Matthew, 2001), with the first artificial intelligence (AI) character traced back to Samuel Butler’s Erewhon in 1872 (Gunn, 1988). Sinophone science fiction has developed its own genre conventions by incorporating aspects of animism which plays a fundamental role in the cultural logic of southeast Asia (Arhem & Sprenger, 2015). Animism involves the idea that, not only animals, but objects and “things” can develop, or have, human-like qualities and that the natural environment is populated by spirits with agency and intelligence (Birrell, 1993). Although mechanoids, such as robots, may seem very different, the traditions of animism allow them to be portrayed as a kind of smart “monster,” who can learn to live in human society and where the boundary between human and non-human, including animals, spirits, and mechanoids, is relatively flexible and innocuous. This makes sense of the close connection between the two mechanoid sex variables and animal sex variables in the Sinophone component structure: a connection conspicuously absent in the Anglophone.

In the Anglophone structure, the two mechanoid sex variables sit unobtrusively in Minority Paraphilia Specialist, while the animal sex variables dominate Animal Sex Specialist. In Christian cultures, the boundary between god, human, animal, and object appears more definite than in southeast Asian beliefs. This may be related to the way in which most Anglophone countries have well-established laws recognizing animals as a special category of being warranting protection (Meiches, 2018). In fact, animal rights are a basic ethical principle in Anglophone countries and has a long legal history (Vallentyne, 2005). Non-human humanitarianism is paid less attention in Sinophone cultures, and Chinese, Japanese, and Korean authorities have few regulations with regard to livestock and pets (Trent et al., 2005). Moreover, human–animal sex is strictly taboo in Christianity (Aggrawal, 2009) and is illegal in most Anglophone countries, and the representation of real sex between humans and animals is illegal under some extreme pornography legislation (Wilkinson, 2011).

In contrast, although illegal in Hong Kong (Cap 200 s 118L Bestiality [CRIMES ORDINANCE]), sex with animals is unregulated in Mainland China and discussion of this topic is not allowed in mainstream media. In BL stories, animal sex is rarely portrayed as intercourse with an actual animal. More usually, characters present with only some animal features, or morph between human and animal form. However, cultural taboos might still be implicated for an Anglophone audience. Hence, the strict boundary between human and animal, and the long history of science fiction portrayal of mechanoids, may account for the distinction found in the Anglophone data between animal sex as a major specialism and mechanoid sex as one of many diverse minority paraphilias.

#### The Variable Tentacle Sex

For Sinophone BL fans, tentacle sex appears in BDSM Specialist. Tentacle sex is a mainstay of Japanese erotic arts (Uhlenbeck & Winkel, 2005). Although it is rare for there to be explicit sexual interludes in Japanese and Chinese myths and folktales, animism provides a wide range of opportunities for imagining sex between humans and eagerly engaging animals and other beings. Hence, in tentacle sex the octopus is easily understood to be using their body erotically and this motivation is sometimes extended to sinewy objects such as branches and lianas in danmei stories as part of BDSM power-play. On the other hand, for Anglophone BL fans, tentacle sex sits in the Minority Paraphilia Specialist component possibly due to lack of familiarity with this kink trope.

### Sub-Demographic Groups

Although Minority Paraphilia Specialist engaged a large enough group in the Sinophone sample to identify demographic patterns, none were found. This makes sense given that the component, almost by definition, consists of individuals with a variety of different niche sexual interests. However, in relation to the other components, in both cultural groups it is the avid BL fans and/or those who like explicitly sexual stories who are most engaged (see also with regard to incest themes, Madill & Zhao, 2021b). Fandom intensity and the liking for explicitly sexual stories correlate strongly in both groups indicating that this is largely one and the same demographic. Zsila et al. (2017) report that being aroused and sexually titillated by the material is a major motivation for engagement with BL. Our study adds that although many may engage with BL out of sexual curiosity, ardent fans may be fulfilling paraphilic interest because differentially it is they who particularly like the sexual content and engage with the more risqué content. This is broadly in line with research showing that high sex drive (Dawson et al., 2016), and preoccupation and hypersexuality (Yakeley & Wood, 2014) are associated with paraphilic interests.

Gender and sexual orientation differentiated Sinophone, but not Anglophone, participants engaging with risqué content captured by the largest three components such that they were more likely to be other-gendered and non-heterosexual. We found a similar, though less consistent, pattern with regard to engagement with incest themes in BL (Madill & Zhao, 2021b). The Anglophone BL audience is predominantly non-heterosexual, whereas the Sinophone is predominantly heterosexual and has a lower proportion of other-gendered people and of avid fans (Madill & Zhao, 2021c). This might explain why there is an association between both other-gender and non-heterosexual orientation and liking of risqué content only for the Sinophone participants. China maintains a conservative society in which mainstream morality does not condone explicit sexual material, and family values play a fundamental role in daily life (Wang & Zeng, 2009). Gender- and sexually non-conforming people may be less likely to align themselves with such values and to explore with intensity diverse sexual material (Wan, 2013). Chinese social conservativism may also account for women’s proportionally lower engagement with risqué BL content given the particularly stigmatizing social implications for them compared to men.

The one exception is that, alongside the patterns discussed above, Sinophone Underage Sex Specialists are also particularly likely to be male. Young characters in BL generally present less masculine gender traits in both visual and narrative forms, having a small, androgynous body and innocent demeanor (Pagliassotti, 2010). Hence, in a strongly patriarchal society, such as China, boy characters may be attractive to men because they can echo traditional gender roles or fantasy of pederastic bonding and mentorship. For other-gendered Sinophone readers, the androgynous presentation of boys in danmei may resonate with an ideal-self and/or gender non-conforming social positioning.

Concern about legal issues was relatively low in both groups. This offers some evidence for the credibility of our data given there appears to be little fear of self-incrimination, although we found a consistent small effect for legal issues with the Sinophone group on incest material (Madill & Zhao, 2021b). Similarly, Sinophone participants who like risqué content demonstrate more concern about legal issues compared to Anglophone participants in all four components, while this reached statistical significance only for Mechanoid/Animal Sex Specialist**.** This specific result is difficult to interpret. In Mainland China, there are rigorously enforced restrictions on sexually explicit materials (Bao, 2020) which may explain the greater concern about legal issues in the Sinophone group. However, risqué content is no more illegal than other danmei material and so there is little reason for Sinophone participants to treat the risqué content questions as particularly sensitive. While there is no room for complacency, legal restrictions on virtual sexualized images of children vary across the Anglophone world, fans may not view BL characters as children, and the clearly imaginary portrayal of human–animal sex in BL, for example, does not contravene extreme pornography laws, at least in the UK (Madill, 2015; Steely et al., 2018) (Fig.1)

.

## Conclusions

Based on the largest data sets in the field, our study identifies a robust pattern of paraphilic interests served through the medium of female-oriented male–male erotica in two contrasting regions of world importance. We have demonstrated for the first time that it is the largely overlapping demographic of avid BL fans and those who enjoy sexually explicit material who tend to consume the risqué sexual content, while, for the Sinophone, this includes also more of non-heterosexual and/or other-gendered people. Hence, we believe it is fruitful to consider interest in male–male sexuality a common, yet overlooked, female paraphilia through which women with intense and frequently engaged atypical sexual interests explore a range of well-known paraphilic themes. Importantly, the forms in which BL manifest, i.e., writing, artwork, and popular entertainment, might indicate why women’s paraphilias may have been largely overlooked. That is, they might be expressed more commonly through fantasy than action, that their mass expression has awaited both the means and the market force, and that current conceptualization of, and assumptions about, paraphilias is overly modeled on that of men.

## Data Availability

Please contact the corresponding author.
